# Evaluation of the Ghana Heart Initiative - Design and Rationale of a Pragmatic Mixed-Methods Study from Diverse Perspectives: A Study Protocol

**DOI:** 10.21203/rs.3.rs-2893313/v1

**Published:** 2023-05-15

**Authors:** Kavita Singh, Elom Otchi, Rupal Shah-Rohlfs, Emilia Udofia, Valérie R. Louis, Isaac Adomako, Nana Ayegua Hagan-Seneadza, Nikias Herzhauser, Afua Boatemaa Owusu, John Tetteh, Daniel DeGraft-Amoah, Eugene Kallson, Volker Franz Winkler, Alfred Edwin Yawson, Manuela De Allegri

**Affiliations:** Heidelberg Institute of Global Health; Korle Bu Teaching Hospital; Heidelberg Institute of Global Health; University of Ghana; Heidelberg Institute of Global Health; University of Ghana; University of Ghana; Heidelberg Institute of Global Health; University of Ghana; University of Ghana; University of Ghana; IQVIA (Middle East & Africa); Heidelberg Institute of Global Health; University of Ghana; Heidelberg Institute of Global Health

**Keywords:** Hypertension, Cardiovascular disease, Ghana, Implementation research, Quality of care, Health systems strengthening, RE-AIM evaluation framework

## Abstract

**Background:**

Rigorous evaluations of health system interventions to strengthen hypertension and cardiovascular disease (CVD) care remain scarce in sub-Saharan Africa. This study aims to evaluate the reach, effectiveness, adoption / acceptability, implementation fidelity, cost, and sustainability of the Ghana Heart Initiative (GHI), a multicomponent supply-side intervention to improve cardiovascular health in Ghana.

**Methods:**

This study adopts a mixed- and multi-methods design comparing the effects of the GHI in 42 intervention health facilities (i.e. primary, secondary and tertiary) in the Greater Accra Region versus 56 control health facilities in the Central and Western Regions. The evaluation design is guided by the RE-AIM framework underpinned by the WHO health systems building blocks framework, integrated by the Institute of Medicine’s six dimensions of health care quality: safe, effective, patient-centered, timely, effi cient, equitable. The assessment tools include: (i) a health facility survey, (ii) a healthcare provider survey assessing the knowledge, attitudes, and practices on hypertension and CVD management, (iii) a patient exit survey, (iv) an outpatient and in-patient medical record review and (v) qualitative interviews with patients and various health system stakeholders to understand the barriers and facilitators around the implementation of the GHI. In addition to primary data collection, the study also relies on secondary routine health system data, i.e., the District Health Information Management System to conduct an interrupted time series analysis using monthly counts for relevant hypertension and CVD specific indicators as outcomes. The primary outcome measures are performance of health service delivery indicators, input, process and outcome of care indicators (including screening of hypertension, newly diagnosed hypertension, prescription of guideline directed medical therapy, and satisfaction with service received and acceptability) between the intervention and control facilities. Lastly, an economic evaluation and budget impact analysis is planned to inform the nationwide scale-up of the GHI.

**Discussion:**

This study will generate policy-relevant data on the reach, effectiveness, implementation fidelity, adoption / acceptability, and sustainability of the GHI, and provide insights on the costs and budget-impacts to inform nation-wide scale-up to expand the GHI to other regions across Ghana and offer lessons to other low- and middle-income countries settings as well.

**RIDIE Registration Number:**

RIDIE-STUDY-ID-6375e5614fd49 (https://ridie.3ieimpact.org/index.php).

## Background

Cardiovascular diseases (CVD) are the leading cause of death globally, with a disproportionately higher burden (> 80% age-standardized CVD deaths) in low-and middle-income countries (LMICs) [1]. Arterial hypertension, also called high blood pressure (BP), is a leading modifiable risk factor associated with CVD death and disability [2]. While the average age-standardized BP is declining in most high-income countries, it is increasing in LMICs with 32%−50% of adults estimated to have high BP in sub-Saharan Africa (SSA) [3]. Multiple studies have shown that the key drivers of CVD in LMICs include insuffi cient physical activity, low fruit intake, high consumption of processed meat and sedentary lifestyles associated with rising urbanization as well as rising prevalence of hypertension, diabetes, obesity, and smoking [4] [5]. Although hypertension is a largely controllable condition, its actual rates of awareness, treatment, and control are disappointingly low in SSA. Ghana is a lower middle-income country in SSA with an estimated hypertension prevalence of 38%, and sub-optimal hypertension awareness (34%), treatment (32%) and control rate (2%−12%) [6] [7] [8]. A 5-year review of autopsy cases (January, 2006 to December, 2010) at Korle Bu Teaching Hospital, the largest and leading supra-tertiary level national referral and teaching hospital in Accra has attributed 22.2% of all deaths to CVD [9]. In addition to the disease burden, CVD threatens to impose a significant economic burden in Ghana, with more than 40–50% of households paying out-of-pocket for health services and medications [10]. Although there is a National Health Insurance Scheme, it has traditionally covered outpatient visits to improve access for many people, with recent expansion to inpatient care coverage although some services at tertiary level hospitals are not included in the benefit package. Additionally, there are delays in reimbursement of claims to facilities that affect the health system’s ability to provide timely management of hypertension and CVD [11].

Evidence from meta-analysis of randomized trials have shown that lowering BP reduces the risk of future stroke by 35%−40% and myocardial infarction by 20%−25% [12]. However, uptake of proven CVD therapies is sub-optimal globally such that there is a 55%-point gap in the effi cacy of CVD treatment shown in clinical trials versus real world effectiveness [13] [14]. Several health system factors in the LMIC context ostensibly influence the poor uptake of CVD prevention therapies and access to care, including the lack of standardized CVD treatment guidelines, acute shortage and inequitable distribution of trained health care workers, insuffi cient health system infrastructure and distribution of health care facilities, profound lack of laboratory facilities, supplies, and equipment including sphygmomanometer, medication stock-outs and high treatment costs [15]. The management and prognosis of high-risk patients depend on well-staffed emergency rooms and critical care units, which are poorly designed and developed in SSA, including Ghana. Further, recent reports from Ghana indicate large gaps in provider training, patient education, and medication availability to manage hypertension [16]. Given the rising CVD burden and relatively lower rates of hypertension control in Ghana, well-designed innovative models of healthcare delivery are urgently needed for early detection, treatment and follow-up of hypertension and CVD [17].

The Ghana Heart Initiative (GHI) initiated in September 2018, and offi cially launched on 9th January 2019, introduced an innovative health system intervention to strengthen early detection, and management of hypertension and CVD across health facilities in the Greater Accra Region (GAR). The initiative was implemented as a collaborative effort by the Deutsche Gesellschaft für Internationale Zusammenarbeit (GIZ) GmbH, Ministry of Health (MoH) and the Ghana Health Services (GHS). The GHI (“the intervention”) enables frontline healthcare workers to better address the needs of patients and communities and support advocacy and policy that promotes effective care for CVD. The intervention does not target the single patient but changes the quality of care given to the single patient, by changing the way the health system addresses the management of risk factors (such as hypertension) and CVD. The overarching goal of this study is to conduct a rigorous evaluation of the GHI, a supply-side intervention implemented in 42 diverse health facilities (primary to tertiary care level) for the prevention and management of hypertension and CVD in the GAR, adopting a mixed-methods implementation science approach.

## General objectives and research questions

Adopting an implementation science approach, our study aims to assess the reach, the effectiveness, the fidelity, the implementation process, the maintenance, and will provide scientific evidence for scaling-up the intervention nationwide. The study is envisioned to provide important insight into barriers and facilitators of scaling up a supply-side quality improvement CVD programme in Ghana. The specific research questions are:

1. Has the Ghana Heart Initiative reached the target population?
2. Has the Ghana Heart Initiative produced the desired changes on quality of hypertension / CVD service delivery i.e., *safety, effectiveness, timeliness, equitable, and patient-centered care* and health outcomes?
3. Has the Ghana Heart Initiative obtained support from key stakeholders?
4. Has the Ghana Heart Initiative been delivered as planned (fidelity)?
5. Are the implementation approach and health system changes produced by the Ghana Heart Initiative sustainable?

## Methods

### Study Setting

Ghana is a lower-middle-income country with an average annual per-capita gross domestic product of US$2,445.3 [18]. The mortality from coronary heart disease in 2020 was 119.93 per 100.000 population, and the probability of dying from any of CVD, cancer, diabetes and chronic respiratory disease is 20.8% [19]. The health system in Ghana is decentralized, pluralistic, and operates on an integrated three-tier (national, regional, and district) scheme and incorporates a community-level health delivery system to support community-based primary healthcare [20]. Most healthcare is provided by the government and is largely administered by the service delivery agencies under the MoH such as the Ghana Health Service (GHS), teaching hospitals, and the private providers (self-financing, faith-based, and non-governmental organisations). Ghana reformed its National Health Insurance Scheme in 2003 with 40% of the population being enrolled and ~ 60% enrolled people are exempted from premium payments [21]. Ghana is one of the first African nations to enact legislation and earmark financing for universal health coverage; a significant move towards health system strengthening and improved financial protection [21].

Accra is the capital city of Ghana and is located in GAR, which is one of the 16 administrative regions, and has the highest population density. The GAR has 26 districts, including the Accra Metropolitan Assembly, and had a total population of 5.4 million in 2021. The pilot phase of the GHI was implemented in 42 health facilities located in five districts (Accra Metro, Tema Municipal, Ga East, Ga West, Dangme West and Dangme East). A health facility is an infrastructure built and licensed by the Health Facilities Regulatory Authority to take care of the healthcare needs of the citizens of Ghana.

**Figure 1:**
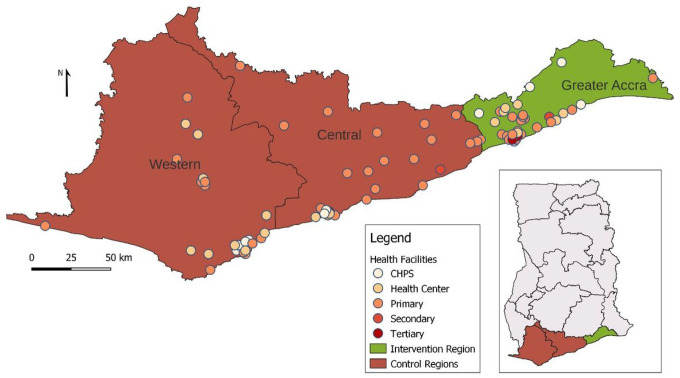
Intervention and Control Regions to Evaluate the Ghana Heart Initiative CHPS = Community Health and Planning Services = Rural Primary Health Care Facilities

The comprehensive evaluation is being conducted in those 42 health facilities implementing GHI with matched control health facilities. Matching of intervention and control facilities was done based on pre-determined variables such as the level of care (primary, secondary, or tertiary), volume of outpatient visits, bed capacity and availability of human resources (number of physicians, nurses, and other allied healthcare professionals). The data on the facility level characteristics was obtained from the GHS and the Christian Health Association of Ghana (CHAG) Secretariat (the largest faith-based health service provider in Ghana) and we considered a large pool of potential ‘control’ health facilities from the Central Region and Western Region (Fig. 1). The advantage of using a quasi-experimental design with matched control facilities ensures that all the exposure variables stay the same across the intervention and control facilities except for the intervention arm, i.e., GHI-related activities.

### Study intervention

The first phase of the GHI, a three-year (2018–2020) programme funded by Bayer AG and implemented through the GIZ, was designed to improve risk assessment and management of hypertension and CVD in public health facilities and hospitals in the GAR. Its primary objective was to improve prevention, detection or diagnosis, and management of CVD. The GHI includes five main components: (i) Stakeholder engagement and development of national guidelines for the management of CVD, (available in hard copies and a digital app); (ii) Training of healthcare workers coupled with coaching / mentorship visits; (iii) Equipment supplies for the diagnosis and treatment of CVD; (iv) Training of healthcare workers for the collection of facility-level data for programme monitoring and the GHI programme team worked with the Policy, Planning, Monitoring and Evaluation Division of the GHS to include important NCD indicators including CVD in the District Health Information Management System (DHIMS2) to facilitate facility-level routine data collection and reporting; and (v) Establishment of a round the clock (“24/7”) CVD support center.

### Conceptual framework

In line with the abovementioned implementation science approach and the aforementioned research questions, our study adopts the RE-AIM framework as the guiding operational evaluation framework. The RE-AIM framework offers a comprehensive structure that will guide the quantitative and qualitative data collection to systematically evaluate the robustness of the intervention (“the GHI”) across the RE-AIM domains – reach, effectiveness, adoption, implementation, and maintenance as well as related costs, and the potential for scaling up the intervention to other regions in Ghana [22]. It should be noted, that within the effectiveness domain, some of the indicators may have elements to estimate impact, where we hope to carry out a Difference-in-Difference (DID) analysis and if routine health data is available an interrupted time series-analysis (ITSA) enabling a quasi-experiment to attribute causality to the intervention.

The Theory of Change (ToC) development was informed by a discussion with stakeholders of how the intervention (“the GHI”) induced changes in the management of hypertension and CVD care in a “real-world setting”, a description of the health systems pathway through which this change was expected to happen, and the RE-AIM evaluation framework to assess whether and how change has happened [23] [24].

Considering the content of our intervention specifically, we turned to the World Health Organization’s (WHO) six health system building blocks (HSBB) to structure our work [25]. We did so out of a desire to analyze systematically how the actions carried out by the GHI had produced effects on each of the six HSBB. The WHO HSBB at health facilities enables a comprehensive evaluation of the health system performance, clinical, processes of care and quality of care outcomes. Further, understanding implementation outcomes (fidelity, acceptability, and effectiveness) of the intervention using a conceptual ToC model from diverse user’s perspectives can support consideration of how and why the intervention is likely to generate the changes observed and assist in thinking through the potential for the expansion and scale-up of the GHI in contextually similar settings. It may also contribute to consideration of how to manage implementation over time and highlight factors that are likely to support the sustainability and achievement of long-term goals of the GHI.

To ensure measures of patient experience of healthcare quality are valid (measuring what they intend to) they need to capture a wide array of domains. Since the core of the GHI focused on enhancing service delivery models, we further integrated the widely accepted six domains of health care quality by the Institute of Medicine (IOM) into our conceptual framework. We thus, look at the six domains; care which is Safe, Timely, Effi cient, Effective, Equitable and Patient-centered, referred to as ‘STEEEP’ [26]. Taking ‘timely’ as an example; in an outpatient setting, timely might be the length of time a patient has to wait for his/her CVD consultation, whereas in a hospital setting, it may be interpreted as the length of the time the patient has waited for a diagnostic test.

The major activities that the GHI focused on are all believed to support the health system attain its mission and vision: quality health service delivery, leadership and governance, and capacity building of the health workforce. Thus, evaluation of the overall Ghanaian healthcare system in the context of implementing the intervention using the HSBB (service delivery, health workforce, medicines/vaccines and technology; health information systems; financing; and leadership/governance) is required to identify gaps as well as provide insights to further scale up and sustain the activities of the GHI across Ghana. In this evaluation, emphasis is placed on the interaction and interdependence across and within building blocks from a health system perspective. We will also look at how the demand side of health services (community and patients) interacts with the intervention components given the context in which the intervention is being implemented and to assess effectiveness, safety, and timeliness of patient-centered care. We hope to demonstrate the pathways through which the GHI operates to achieve the desired outcomes (i.e., improvements in screening, diagnosis and control of hypertension and CVD).

**Figure 2:**
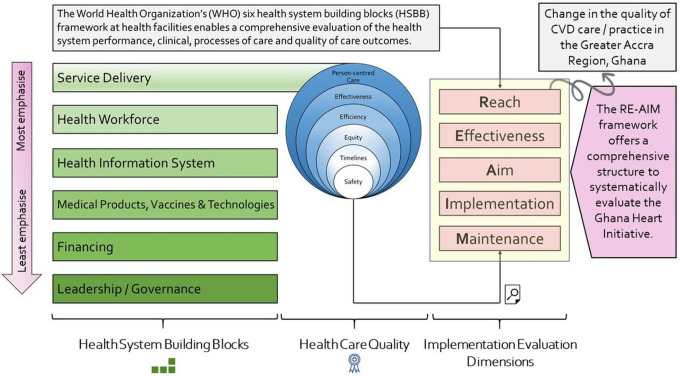
Theory of Change that Underpins the Evaluation of the Ghana Heart Initiative

**Figure 3:**
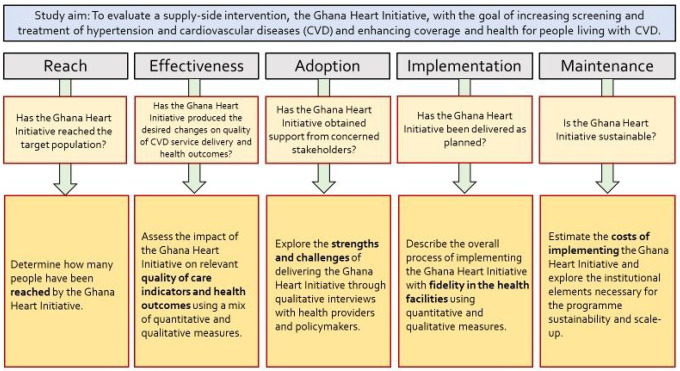
Overview of the RE-AIM Evaluation Framework

Figure 2 provides a visual illustration of our conceptual framework while Fig. 3 outlines the specific research questions addressed under each dimension of the RE-AIM framework, being informed by the conceptual understanding of the HSBB and the IOM’s STEEEP quality framework described above.

### Study design

This study adopts a triangulation mixed- and multi-methods quasi-experimental design. A triangulation mixed methods design employs quantitative and qualitative methods in parallel, holding equal weight within a given research context, and appraised jointly to understand complex phenomena and changes. In this case, the authors investigate changes inducted by the GHI. A multi-method design involves multiple sources of data collection and/or relies on multiple analytical strategies, within each strain of work, the quantitative and the qualitative. The evaluation research protocol development was informed by wide consultation, including review by the research teams at the University of Ghana Medical School, Ghana, and the Heidelberg Institute of Global Health (HIGH), Germany. Researchers took part in a study design workshop in Accra in April 2022. In addition, between April and July 2022, multiple virtual meetings were organized to agree on the final study design and data collection protocol.

### Study components

**Figure 4:**
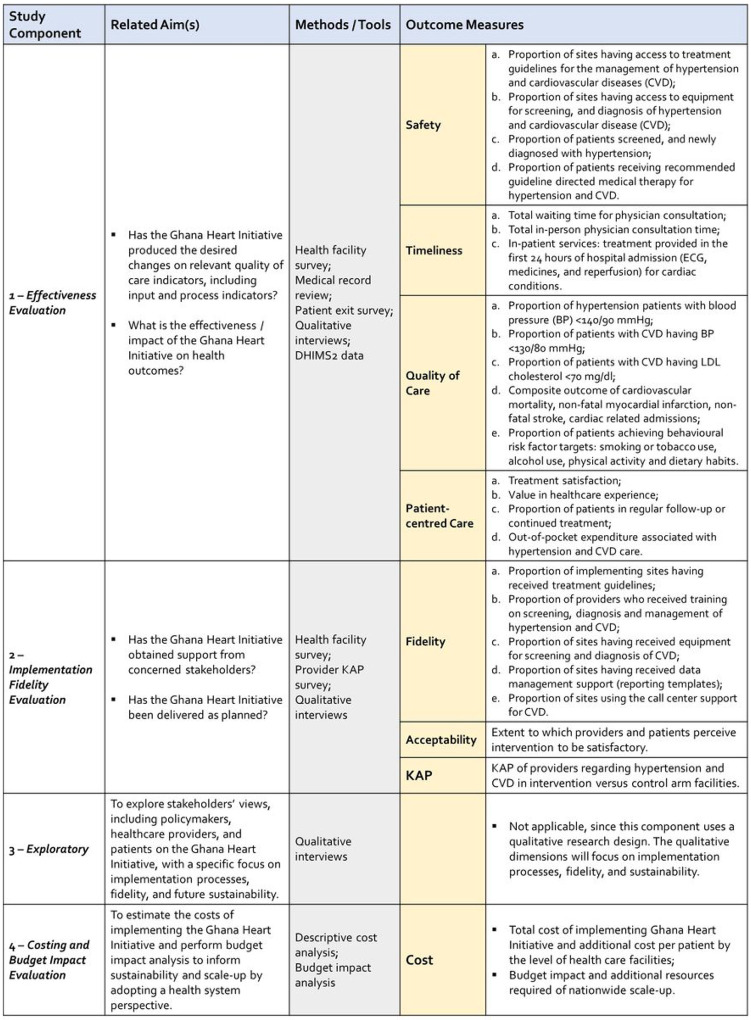
Summary of the evaluation components, tools and outcomes measures *Fidelity and Acceptability were only assessed in the interventional health facilities. KAP = Knowledge, attitude and practice.

This is a facility-based study, meaning that, with the exception of the interviews with policy makers and mid to high-level health system offi cers, all other data collection activities take place at the facility level. In all participating health facilities, we implement multiple data collection tools: (1) Health facility survey; (2) Healthcare provider survey to assess knowledge, attitudes, and practices (KAP) on hypertension and CVD; (3) Patient exit survey; and (5) Medical record review (MRR) before and after implementing the GHI. In addition to primary data collection, the study also relies on secondary routine health system data obtained by the study team in fully anonymized format, the DHIMS2. Figure 4 provides a summary of the health service delivery evaluation domains, measures, or indicators (process, outcomes, and sustainability), and tools.

### Quantitative study component

For the assessment of the health service delivery performance between the intervention and control facilities, we are utilising mixed methods and multiple survey tools. Individual methods are chosen strategically based on the research question as well as considering feasibility and practicality [27]. Data is being collected at the facility-, provider- and patient-level using multiple surveys:

1. **Health facility assessment survey**: to determine the existing infrastructure, human resources, availability of services for hypertension and CVD, availability of laboratory tests and diagnostics, medicines for hypertension and CVD treatment and health service delivery indicators related to hypertension and CVD.
2. **Provider survey**: to examine the knowledge, attitudes, and practices (KAP) regarding CVD and hypertension management as well as the acceptability of the GHI from provider’s perspective in intervention facilities only.
3. **Patient exit survey**: patients asked post-consultation about the provision of care in the consultation to assess patient experiences and satisfaction with the hypertension or CVD related care received at the intervention and control facilities.
4. **Medical record review**: before and after the implementation of the GHI to assess differences in hypertension and CVD care practices between intervention and control facilities.

For the provider KAP survey and exit survey, the field data collection team(s) visit a health facility and capture a random sample of healthcare providers and patients available on the day of the visit. This method relies on random sampling and the effi ciency of the field team. In essence this means maximizing the number of potential respondents to be interviewed on the day of the visit i.e. all eligible patients exiting the facility are interviewed, all eligible healthcare providers present and available at the facility are interviewed [28] [29]. The field work is carefully timed so that it coincides with the day of a hypertension / CVD clinic. Prespecified **eligibility criteria** is applied for health facilities and study participants (providers and patients) for the quantitative evaluation. The health facility assessment survey is carried out in all GHI intervention facilities, and matched control health facilities to specific characteristics (example, level of care, volume of patients attending the clinic, bed size, human resources available, etc.). The provider survey is recruiting providers (doctors, nurses, physician assistants, pharmacists) aged 18 years and above, both males and females; presently working in the facility at least from the last 3 months. Further, a licensed provider is defined as a physician or a nurse, pharmacist or physician assistant who is directly involved in providing CVD related services (counseling, health education, or consultation services or pharmacological therapy). The provider survey excludes those healthcare workers who have a managerial position and do not provide any patient care. Also, a physician, nurse or pharmacist who has worked in the facility for less than 3 months is excluded from the provider survey. The patient exit survey is carried out among those aged 18 years, both males and females, and having physician diagnosis of hypertension or other CVD conditions. The exclusion criteria include patients having diseases associated with frequent hospitalization (advanced cancer, end stage renal disease) bedridden or debilitating conditions. The MRR is performed for the patient folders for those aged 18 years and above, both males and females, and having physician diagnosis of hypertension or CVD conditions-Medical records from 2018 to 2022 are being reviewed. The medical records of pregnant women and those patients having diseases associated with frequent hospitalization (advanced cancer, end stage renal disease); bedridden or debilitating conditions are excluded for the MRR.

### Data collection Tools, Indicators, and Strategies

The quantitative evaluation component is critical to learning whether intervention improves outcomes among patients seeking care for hypertension and CVD in health care facilities. This component includes the following sets of quantitative tools, specifically:

1. **Facility based structured survey**, including analysis of facility registries & clinical records, to capture indicators of quality-of-service delivery (input, process, outcome).
2. **Interviews with healthcare providers** to assess the knowledge, attitude, and practice regarding hypertension and CVD.
3. **Exit interviews with the patient** to assess the client’s perception of the CVD consultation or examination, as well as his / her recollection of the instructions that he / she received about treatment or preventative behaviour.
4. **Review of medical records** to capture indicators of quality-of-service delivery before and after GHI implementation.
5. **Health management information systems** data with data being aggregated monthly at facility level for each of the CVD outcomes of interest.

The rationale for selecting service delivery, processes of care, clinical and quality of care indicators are based on the aspirations to achieving optimal health service performance and to guide the health care planning as a continuous quality improvement tool to drive health service improvement and better health outcomes [30] [31]. The outcome indicators are chosen from the recommendations of the national and international guidelines focused on CVD as well as to reflect how the facility or provider level determinants affect individual health behaviour and wellbeing (Table 1).

The exit survey draws questions from previous patient exit and household surveys and were further adapted to fit the Ghanaian context. Demographic and socioeconomic questions were based on the Ghana Census and Demographic and Health Survey. The exit interviews with the patient examines the respondent’s perception of the CVD consultation or examination, as well as his/her recollection of the instructions that he/she received about treatment or self-care behavior. The survey also captures how patients perceive the quality of specific aspects of the health facility and its providers. Patients are asked to rate 27 aspects of the facility visit on a five-point Likert scale with related Emoji’s.

The medical records of outpatient and inpatient folders at two cross-sectional timepoints before and after GHI implementation are being reviewed to assess between group differences in hypertension and CVD care delivery. Eligibility of medical records are pre-specified based on confirmed diagnosis of hypertension or CVD, and complete medical records with at least data available on patient demographics (age, sex), medical history (confirmed case of hypertension or CVD), laboratory results (any test prescribed, or test reports reviewed), and medical treatment or prescription.

Lastly, DHIMS2 data routinely collected by the MoH in Ghana to monitor service provision consisting of monthly facility-specific counts for a wide range of services, including CVD will be analyzed to assess the differences in care delivery between the intervention and control health facilities over the time period. In line with prior work, to translate monthly counts into indicators that adequately capture service coverage, we construct outcome variables that account for the underlying target population living in the catchment area of a given facility [32]. More specifically, we apply interrupted time series analysis to DHIMS2 data to assess impact on the following CVD indicators: prevalence of hypertension, coronary heart disease, stroke, heart failure and related mortality.

### Study outcomes

The primary outcome measures being assessed between the intervention and control facilities are: i) proportion of screened individuals for hypertension, ii) newly diagnosed patients with hypertension or CVD, iii) prescription of guideline directed medical therapy for hypertension and CVD, iv) BP control defined as < 140/90 mmHg, v) provider knowledge and practice related to hypertension and CVD and vi) patient centered care and satisfaction. The study also assesses several secondary outcome measures including difference in total waiting time for physician consultation and physician acceptability of the GHI.

### Sample size

The sample size of 1,200 participants in total for the MRR or patient exit survey will provide 90% power to detect 10% relative difference between the intervention and control facilities in processes of care indicators such as prescription of evidence-based therapies and blood pressure control. at an α = 0.05 and accounting for 20% non-response rate or missing data.

### Statistical analysis

Quantitative data is analyzed by descriptive statistics for demographic, socio-economic and other patient-, provider- and health system level factors between the intervention and control facilities. Differences in service delivery indicators, process of care measures (prescription of guideline directed medical therapy) and clinical outcomes (BP control) will be compared between the intervention and control facilities. The chi-square test is used to evaluate differences between categorical variables. Two-sided p-values < 0.05 will be considered statistically significant. Composite performance scores are defined by determining the proportion of patients who receive concordant care compared to patients who receive discordant care between the intervention and control facilities. Linear and logistic regression analysis techniques are used to assess determinants of health outcomes, processes of care measures, and treatment satisfaction [33]. Random-effects regression analyses will be used to account for cluster effects and imbalances between intervention and control group health facilities; Effect sizes will be presented as risk ratios for binary outcomes, and as mean differences for continuous outcomes; 95% confidence intervals (CI) will be given for both. Missing data in outcome measures will be handled by appropriate imputation methods.

A propensity score will also be created using logistic regression and will contain those variables associated with treatment at a health facility [34]. Analysis of clinical outcomes analysis will also use ANCOVA (analysis of covariance) to compare the prescription of evidence-based medicines, and mean change in BP and lipids between the intervention and control arm facilities [29]. Other outcome measures such as behavioural and biochemical risk factors will be analyzed using logistic or conditional logistic regression to adjust the comparisons for other variables. We shall also compare the percentages achieving clinical and behavioural targets between the intervention and control arm facilities.

The fidelity analysis will uncover the extent to which the GHI was fully or partially implemented by various health system stakeholders.

Next, when analyzing the KAP survey we will report the demographic characteristics of healthcare providers by the intervention and control arm facilities, reporting the mean (standard deviations, SD) or median (25th and 75th percentiles) for continuous variables and frequencies (percentages) for categorical variables. Knowledge about CVD symptoms and modifiable risk factors will be reported as number (percent) by overall and intervention and control arm facilities. Next, each correct response for knowledge related questions will be assigned a score of 1 and the total score for the knowledge of CVD risk factors and treatment recommendations will divided by tertiles and classified as minimal, moderate, and high knowledge. The mean (SD) for knowledge of CVD risk factors and treatment will also be reported by intervention and control facilities. Finally, the correct scores for the knowledge of CVD risk factors and treatment strategies will be classified into high knowledge (> 75% correct responses) and poor knowledge (≤ 75% correct responses) using arbitrary threshold for comparison and also informed by the published literature [35] [36]. The chi-square test will be performed to investigate the difference in knowledge scores between intervention and control arm facilities across age-group, sex, and education strata. Bivariable and multivariable regression analyses will be performed to assess the factors associated with high knowledge of CVD symptoms, and modifiable risk factors. The unadjusted and adjusted odds ratio with 95% confidence intervals (CI) will be reported. The model will be adjusted for age, sex, type of health facility and type of healthcare worker.

Lastly, an interrupted time-series analyses (ITSA) is carried out to establish effects of the GHI on the screening, diagnosis, and management of hypertension / CVD, if aggregate facility-level monthly data are available from the DHIMS2. ITSAs apply segmented regression to assess the impact of events that have occurred at a clearly defined point in time. Data points recorded in the GHI enabled DHIMS2 system will be analyzed by month to examine trends over time and seasonality. We will also evaluate change in patient outcomes over time (e.g., mean systolic BP change).

### Qualitative study component

We propose to conduct qualitative in-depth interviews with policymakers, health administrators, providers, and patients to understand the fidelity, barriers, and facilitators of implementing the GHI in the local healthcare context. For assessing implementation fidelity, semi-structured key informant interviews with policymakers, health administrators and GHI training of trainers are being carried out. Additionally, overall direct and indirect experience with the intervention is explored through in-depth interviews with healthcare providers and patients. Participants are selected purposively, allowing for the researcher to select key informants who will have experience of CVD policy and useful perspectives on the GHI. To enhance the credibility of this sampling, a maximum variation sample is used to ensure the consideration of key demographic variables likely to have an impact on participant’s views, for example, age, and occupation. This aims to ensure that the sample within the selected groups is both diverse and representative of the stakeholders in question, and so maximize a fair share of perspectives and views. Additional participants may be selected using purposive snowball sampling [37]. Data saturation is considered to determine the number of interviews. For conducting the interviews, semi-structured interview guides have been developed for each participant group, and clustered into broad themes to encourage participants to speak freely about their perceptions, experiences and visions. Follow-up questions are prompts and probes aimed at following respondents’ answers and investigating the raised issues more in-depth. In general, the same areas will be covered with every participant so there is some point of comparison, however the topic guide will be adapted for each participant group (in each region). Some participants might be interviewed multiple times. All individual interviews are being audio-recorded and transcribed verbatim by local field researchers and social scientists.

### Qualitative data analysis

Transcripts from individual interviews and key-informant interviews will be analyzed using thematic analysis [38] and will focus on explaining observed heterogeneity and identifying enablers and barriers of the intervention and hypertension and CVD management, with a specific focus on fidelity, implementation and sustainability. We rely on a mixed deductive and inductive approach [39]. An initial set of coding categories are developed based on the themes of the interview guides but will allow additional codes and themes to emerge as we proceed with reading and analyzing the transcribed material. Analyzing data jointly across sources implies that source triangulation is applied [40]. In addition, analyst triangulation [41] is applied since we will ensure that at least two distinct people code and analyze the transcribed material and any discrepancies will be resolved by direct consultation with a third coder. A senior researcher not involved in data collection will review the coding consistency. An expert has given a workshop on the NVivo software to the research staff in order to establish a common platform for the data analyses throughout the study. Data analysis is conducted by the lead qualitative researchers with support from NVivo software version 12.0 (QSR International Pty Ltd, Melbourne, Australia, 2010) [42]. Strategies to collect trustworthy data is guaranteed in the following ways: (1) review the interview guide and coding scheme by HIGH / UGMS core research team; (2) accompany the field researchers during the initial interviews by experienced qualitative researchers (3) review of selected transcripts by senior scientists for triangulation; (4) discussions with and feedback from trained data collectors and evaluation team on emerging themes. Finally, reflexivity includes examining researchers’ own conceptual lens, explicit and implicit assumptions, preconceptions and values and how these affect research decisions in all phases of the qualitative study.

Finally, outcome data is contrasted with implementation data and quantitative and qualitative results obtained from different information sources to validate and complement our findings is triangulated

### Economic evaluation

We are adopting a health system perspective, meaning that costs to enable rolling out the GHI are traced as incurred by the GHS / MoH and its development partner, the GIZ, but excludes costs incurred by the health facilities or patients. Economic costs are estimated but not restricted to financial costs, i.e., the full value of resources being used by any of the parties (MoH; GHS; and GIZ) involved in implementation of the intervention is traced, whether reported in financial statements or not. We rely on Activity Based Costing, i.e., an approach that recognizes the relationship between costs, activities, and products. Accordingly, all activities are mapped and related to the design and pilot implementation of the GHI; then, all resources being consumed by these activities are traced; and finally, all resources being consumed are valued [43].

To collect data on resource consumption and unit costs, we use a mix of financial statements from implementing partners and key informant interviews. The main activities regarding GHI implementation will comprise of: Design, Management, Promotion, Operations research, Monitoring and Evaluation (M&E), Verification, and Supply Side activities. We will ask the implementation team to generate aggregate cost information across specific micro-level activities, which will then be grouped into broader meaningful Cost Categories, and Analysis Cost Categories, to enable drawing a link between Cost Categories and Main Activities [43].

A descriptive cost analysis and budget impact analysis is carried out to inform the nation-wide scale-up of GHI. This may be supplemented with a modelled analysis where the effects and costs are modelled beyond the outcome observed during the study period. This will allow comparative assessment of the value for money that GHI offers compared to usual care. It will also allow comparison with other government initiatives as to their cost-effectiveness in terms of cost per disability-adjusted life year (DALY) averted.

The budget impact analysis is used to identify costs and resources required for offering/expanding the GHI to all patients seeking care at the health facilities in Ghana. The budget impact analysis tracks the total and per participant costs of programme delivery using an activity-based costing (ABC) approach [44]. Using this approach, all relevant labor, materials and supplies, contracted services, travel vouchers and opportunity costs required to deliver the interventions are captured by key activities.

## Discussion

There has been no published scientific evaluation of a multicomponent health system intervention to strengthen hypertension and CVD prevention and control in Ghana, but reviews of studies in other high-income settings show favorable results for guideline-based care and equivocal results regarding training of healthcare workers, and providing equipment support and CVD call center support [31] [45] [46]. Moreover, these mostly small, single-strategy studies with short test periods have tended to focus on intermediate or surrogate endpoints and had limited economic evaluation and sustainability. For these reasons, we sought to comprehensively assess an integrated, and multicomponent CVD risk reduction intervention tailored in Ghana. The GHI introduces integrated care across the healthcare levels by implementing guideline-based prevention, diagnosis, and treatment of hypertension and CVD, training of physician / healthcare worker, supply of equipment for diagnosis and management of CVD, data management support for continuous monitoring and improvement and a CVD call support center operating day and night (“24/7”). Further, the GHI intends to improve the provider’s adherence to guidelines through mentoring/supervisory visits and deliver high quality care to patients with hypertension and CVD.

### Evaluation Design Strengths and Weaknesses

The mixed-methods triangulation study is the first of its kind for LMICs and in sub-Saharan Africa, testing a comprehensive and multifaceted health system intervention addressing hypertension and CVD management. The evaluation is rigorously designed, factoring in multiple data sources, minimizing biases using matched control health facilities, data triangulation using quantitative survey and qualitative interview data, and economic evaluation. The ToC was developed in a participatory manner in collaboration with GHI programme implementers helping the evaluators to understand where the intervention tried to produce a change and led to a commonly defined TOC and definition of indicators. Our focus is centered more on a theory-driven evaluation and the fact that we will work “closer to reality” but far from a real experiment or quasi-experiment. To avoid contamination between the intervention and control facilities, the data collection for control group facilities and participants will be accomplished by a separate team of trained field enumerators. However, in a real-world setting we cannot avoid spillover of intervention components to other regions since the national guidelines for the management of CVD were signed by the Minister of Health in 2021 and offi cially launched in August 2022 by the MoH and is available in the public domain and can be accessed via the AkomaCare App (https://akomacare.org/download) [47]. The data collection timepoints for the MRR before and after GHI implementation has been prespecified in both the intervention and control health facilities to avoid any seasonal variations in the hypertension and CVD care outcomes. Furthermore, we customized the survey questionnaires developed with an open-source platform called Survey Solutions (https://mysurvey.solutions/en/) developed by the World Bank. Data is entered on Android-based tablets using the Survey Solutions Interviewer App, stored locally on the tablets, and synchronized regularly to HIGH’s virtual server, enabling real time tracking and remote data monitoring. The Survey Solutions Designer allows the inclusion of instructions visible in blue for each question as the interviewer progresses through the survey. This digital data collection approach along with its technical features improves data quality and reduces data entry errors or missing values.

The study has several limitations. As health facilities are not randomly assigned to the intervention and control arms, it is possible that the arms will be imbalanced about important patient characteristics (age, disease patterns, severity), provider characteristics (education, professional training or practicing experience), facility characteristics (primary, secondary and tertiary) and ownership (public, quasi-government and faith-based). These would be addressed in analyses adjusted for any important patient or provider-level characteristics. Mixed methods (quantitative and qualitative assessments) data evaluation will complement the overall data triangulation and analytical approach. Another potential limitation of the evaluation process may be recall bias when obtaining interview data after the pilot implementation phase of the intervention, as adherence may vary.

## Conclusions

Independent robust scientific evaluations of supply-side hypertension and CVD care delivery models in LMICs are rare. To date, little research has helped to understand implementation challenges and opportunities that are at the core of complex health system interventions. Moreover, the CVD burden is increasing rapidly in LMICs such as Ghana, and there exists a critical window of opportunity to integrate and deliver high quality systems of care and prevention strategies to ameliorate the CVD burden. In this article, we have presented a comprehensive protocol that aims to study the effectiveness, the fidelity, the implementation processes, the sustainability, the conditions that can influence scaling-up of a CVD care delivery model, the GHI, in a low-resource setting.

## Figures and Tables

**Table 1:** Health service delivery evaluation: domains, indicators, and tools.

Domains	Process Indicators	Outcome Indicators	Sustainability Indicators
Safety	No. and proportion of sites having access to guidelines, equipment; Type of out-patient and emergency services available by facilities.	Annual death rate for CVD; Incidence of CVD; Prevalence of hypertension; Prevalence of CVD	Workforce appropriate (workforce adequate in volume and distribution); Equipment adequate.
Tools	Health facility survey; Key informant Interviews	Health facility survey; DHIMS2 dataset	Health facility survey; Key informant Interviews
Timeliness	Annual hypertension screening coverage; CVD preventive and treatment services offered.	Total waiting time for physician consultations; Total in-person consultation time; Treatment received within 24 hours of CVD admission.	Workforce sustainability (staff retention per year, staff stability per year).
Tools	Health faclity survey	Health facility survey	Key informant interviews
Effectiveness	No. of HTN patients seen; No. of CHD patients seen; No. of stroke patients seen; No. of heart failure patients seen; No. of hospitalizations by CVD conditions; Average length of hospital stays (in days); No. of acute emergency transfers related to CVD; No. of referrals to higher facilities for CVD; No. of referrals to lower health facilities for CVD.	No. and proportion of patient diagnosed with hypertension; Number and proportion of patients diagnosed with CVD; Number and proportion of CVD patients prescribed evidence-based medicines; Number and proportion of patients with BP < 140/90; Number and proportion of patients with LDLc< 100 and < 70 in those with CVD; Number and proportion of patients who do not smoke.	Linkages - referral pathways (coordination of care across providers, specialist access); No of referrals per year; No of specialist consultations per year.
Tools	Health facility survey	Health facility survey; DHIMS2 dataset; Patient exit survey	Health faclity survey; Key informant interviews
Patient centered care	No. of follow-up appointments.	Treatment satisfaction; Proportion of patients in regular follow-up.	Infrastructure (IT-internet access, equipment access, staff availability or shortage)
Tools	Health facility survey	Health facility survey; Patient exit survey; In-depth interviews	Health facility survey; Key informant interviews
Efficiency and budget impact	Cost per service for: Outpatient Visit Emergency Visit Hospital Admission	Equity Proportion of patients receiving care by socio-demographic groups.	Governance and Leadership Description of governance structure and leadership.
Tools	Health facility survey; Patient exit survey	Health facility survey: Patient exit survey	Key informant interviews

* BP = Blood pressure; CHD = Coronary heart disease; OPD = Out-patient department; CVD = Cardiovascular disease; HTN = hypertension; LDLc = Low density lipoprotein cholesterol; HMIS = Health management information system.

## Data Availability

Not applicable for this manuscript as it is a protocol paper and there is no data associated with this paper. Future publications with datasets associated with this evaluation study will become available in accordance with journal policy and/or on request and following acceptance for publication of the main findings.

## Abbreviations

BP: Blood Pressure
CVD: Cardiovascular Disease
DHIMS: District Health Information Management System
GAR: Greater Accra Region
GHI: Ghana Heart Initiative
GHS: Ghana Health Service
GIZ: Deutsche Gesellschaft für Internationale Zusammenarbeit GmbH
KAP: Knowledge, Attitudes, and Practices
LMICs: Low- and Middle-Income Countries
MRR: Medical Record Review
